# Supplementary material for: Crystal structures of Rea1-MIDAS bound to its ribosome assembly factor ligands resembling integrin–ligand-type complexes
Source: Nat Commun. 2019 Jul 11;10:3050. doi: 10.1038/s41467-019-10922-6 (PMC6624252; doi:10.1038/s41467-019-10922-6)
Supplement: Supplementary file 1 — Supplementary Information [file 41467_2019_10922_MOESM1_ESM.pdf]

Crystal structures of Rea1-MIDAS bound to its ribosome assembly factor ligands resembling integrin–ligand-type complexes

Yasar Luqman Ahmed et al.

Supplementary Information

Supplementary Figures

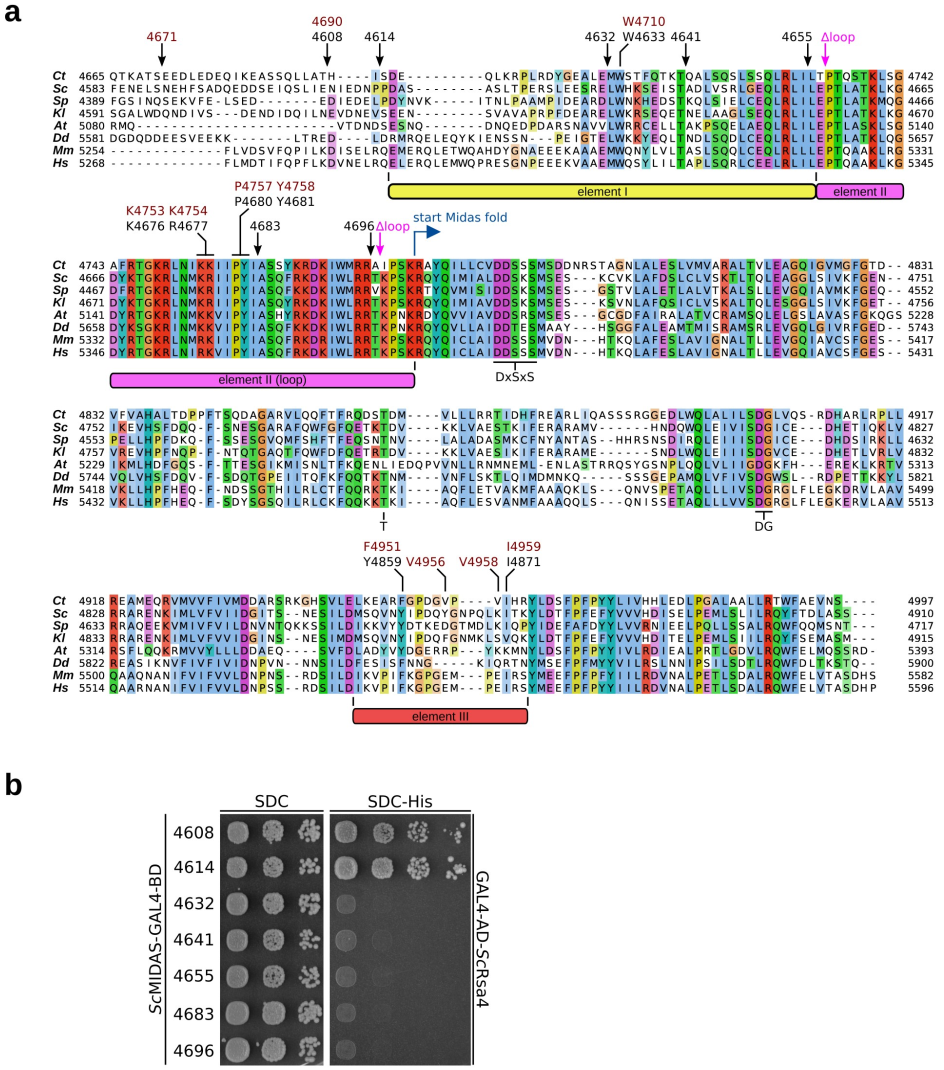

Supplementary Figure 1 Multiple sequence alignment of the Rea1-MIDAS domain and truncation analysis. (**a**) Multiple sequence alignment of Rea1 with homologs showing the MIDAS domain. The sequences of *Chaetomium thermophilum* (*Ct*), *Saccharomyces cerevisiae* (*Sc*), *Schizosaccharomyces pombe* (*Sp*), *Kluyveromyces lactis* (*Kl*), *Arabidopsis thaliana* (*At*), *Dictyostelium discoideum* (*Dd*), *Mus musculus* (*Mm*), and *Homo sapiens* (*Hs*) were aligned with Clustal Omega and visualized with Jalview. The Rea1-specific elements (**I**–**III**) and the residues required for coordination of the Mg^{2+} ion are highlighted below the alignment. Indicated above the alignment are the generated truncations (arrows) and point mutations in *C.thermophilum* (red) and *S.cerevisiae* (black). The boundaries of the Δloop constructs are indicated in pink. (**b**) Yeast two-hybrid analysis of the interactions between the indicated MIDAS constructs with full-length Rsa4 from *S. cerevisiae*. The Rsa4 construct was fused to an N-terminal GAL4-AD (activation domain) and the MIDAS constructs were fused to a C-terminal GAL4-BD (binding domain). The residue numbers indicate the N-terminal boundary of the MIDAS constructs. Plasmids were co-transformed into yeast (PJ69-4A) and transformants were spotted in tenfold serial dilutions on SDC (SDC−Leu−Trp) and SDC−His (SDC−Leu−Trp−His) selective plates. Cell growth was monitored after incubation for 3 days at 30 °C.

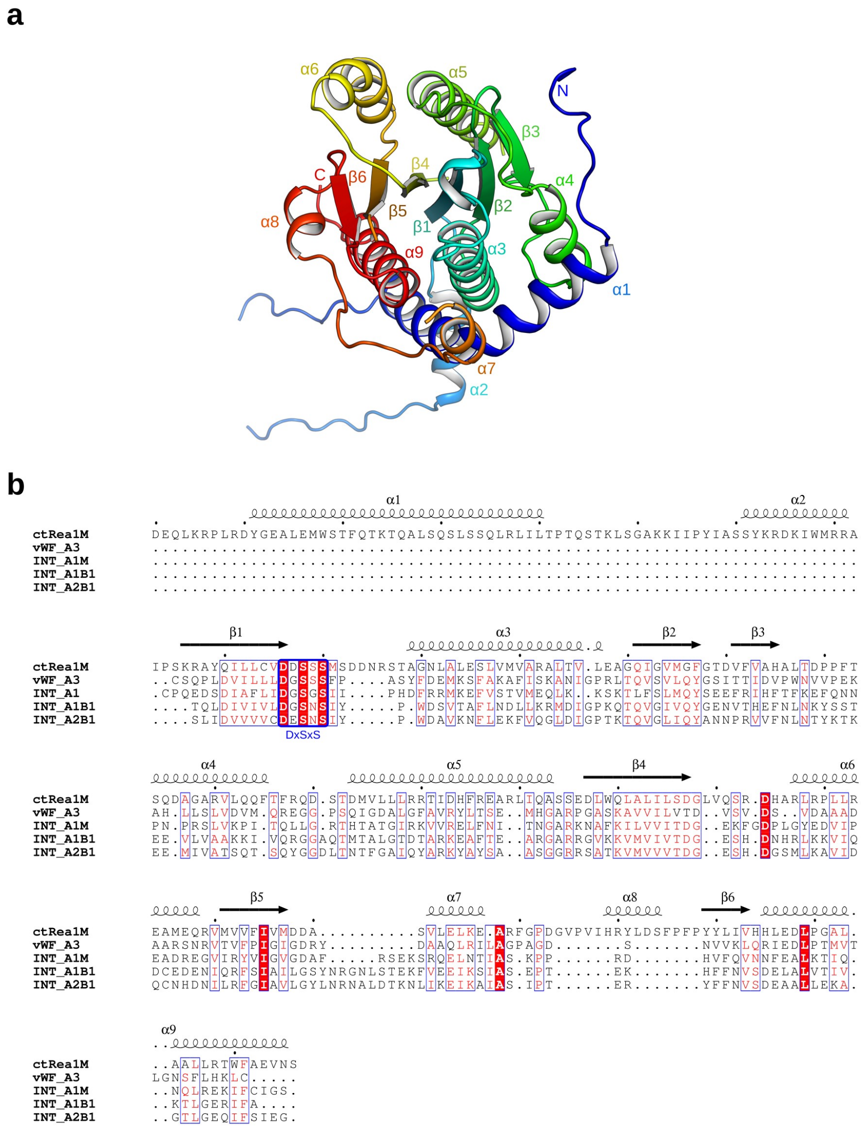

Supplementary Figure 2. Secondary structure comparison of the Rea1-MIDAS and integrin structures. (**a**) Structure of the Rea1-MIDAS domain from *C. thermophilum*. (**b**) Sequence and structure alignment between *Ct*Rea1 and depicted integrin structures. The alignment was created with MAFFTASH. The following structures were aligned: ctRea1M (*C. thermophilum* Rea1-MIDAS, sequence contains gaps not covered in the structure), vWF_A3 (von Willebrand factor A3 domain, PDB ID: 1AO3), INT_A1M (integrin alpha M I domain, PDB ID: 1MF7), INT_A1B1 (integrin alpha1beta1 I-domain, PDB ID: 1QCY) and INT_A2B1 (integrin alpha2beta1 I-domain, PDB ID: 5HJ2).

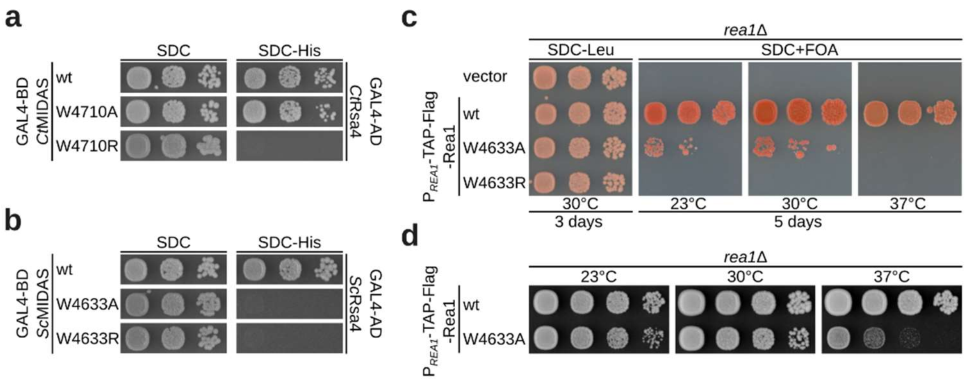

Supplementary Figure 3. Functional analysis of the conserved Try residue in the N-terminal extension of Rea1-MIDAS. (**a**,**b**) Yeast two-hybrid analysis of the Rea1-MIDAS and Rsa4 constructs from *C. thermophilum* (**a**) and *S. cerevisiae* (**b**). The MIDAS constructs were fused to an N-terminal GAL4-BD and the Rsa4 constructs to an N-terminal GAL4-AD. Transformants were spotted on SDC (SDC−Leu−Trp) plates or SDC−His (SDC−Leu−Trp−His) selective plates and cell growth was monitored after incubation for 3 days at 30 °C. (**c**) An empty plasmid control or the indicated Rea1 constructs, fused to an N-terminal TAP-Flag tag, were transformed into a *rea1Δ* shuffle strain. Cells were spotted in 10-fold serial dilutions on SDC−Leu and SDC+FOA plates and cells were grown at the indicated temperatures for 3 and 5 days, respectively. (**d**) Growth analysis of the indicated Rea1 constructs after shuffling on SDC+FOA plates. Cells were spotted in tenfold serial dilutions on YPD plates and growth at the indicated temperatures was monitored after 2 days.

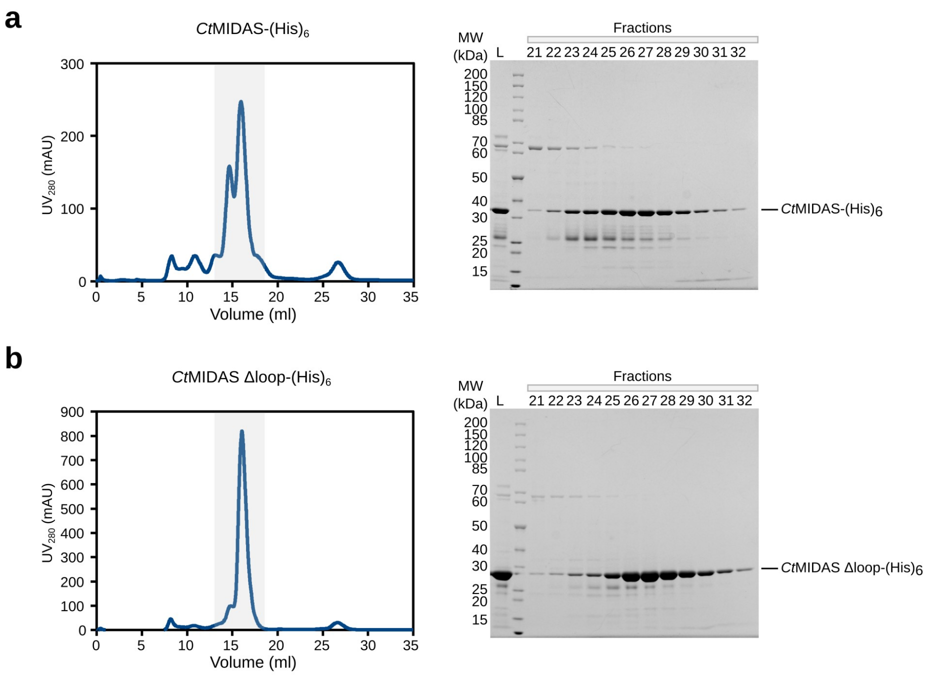

Supplementary Figure 4. Purification of *Ct*MIDAS and *Ct*MIDAS ∆loop. (**a**,**b**) The left-hand panels show the size-exclusion chromatography profiles of *Ct*MIDAS-(His)$_6$ (amino acids 4690-4997) (**a**) and *Ct*MIDAS ∆loop-(His)$_6$ (amino acids 4690-4733-GSG-4774-4997) (**b**). The absorbance at 280 nm (mAU) was plotted against the elution volume (ml). Fractions highlighted in gray were analyzed by SDS-PAGE and Coomassie staining (right-hand panels).

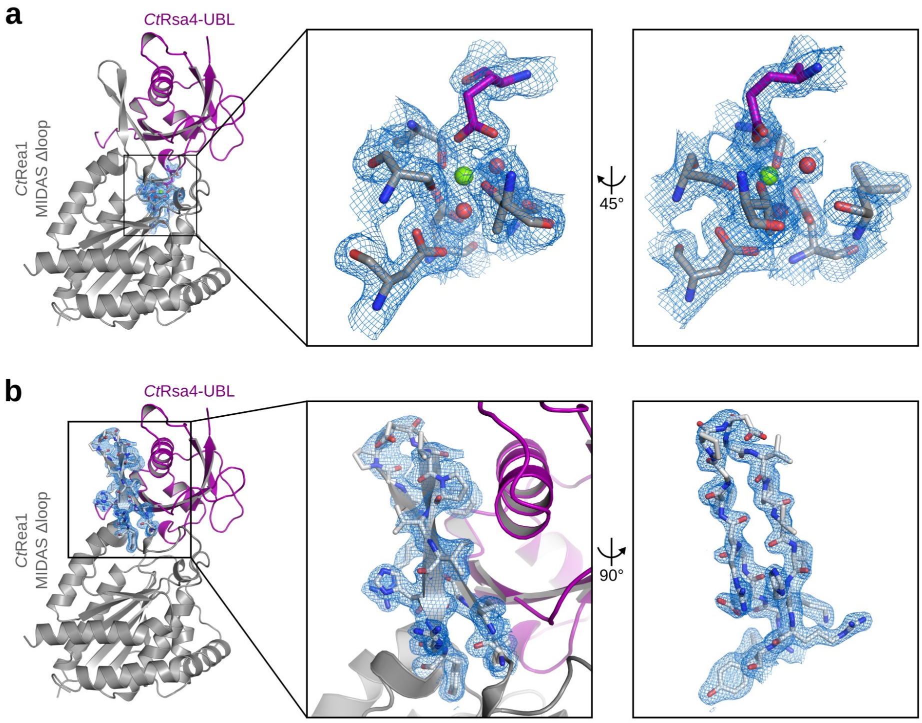

Supplementary Figure 5. Structure of the *Ct*Rea1-MIDASΔloop–*Ct*Rsa4-UBL complex. (**a**,**b**) The *Ct*Rea1-MIDASΔloop–*Ct*Rsa4-UBL model and corresponding electron densities of the Mg^{2+} coordination site (**a**) and the β-hairpin of the Rea1-specific element III (**b**) contoured at 1.5 and 1.0 sigma, respectively. The *Ct*Rsa4-UBL is highlighted in purple and the *Ct*Rea1-MIDASΔloop is shown in gray. Close-up views are shown in two orientations.

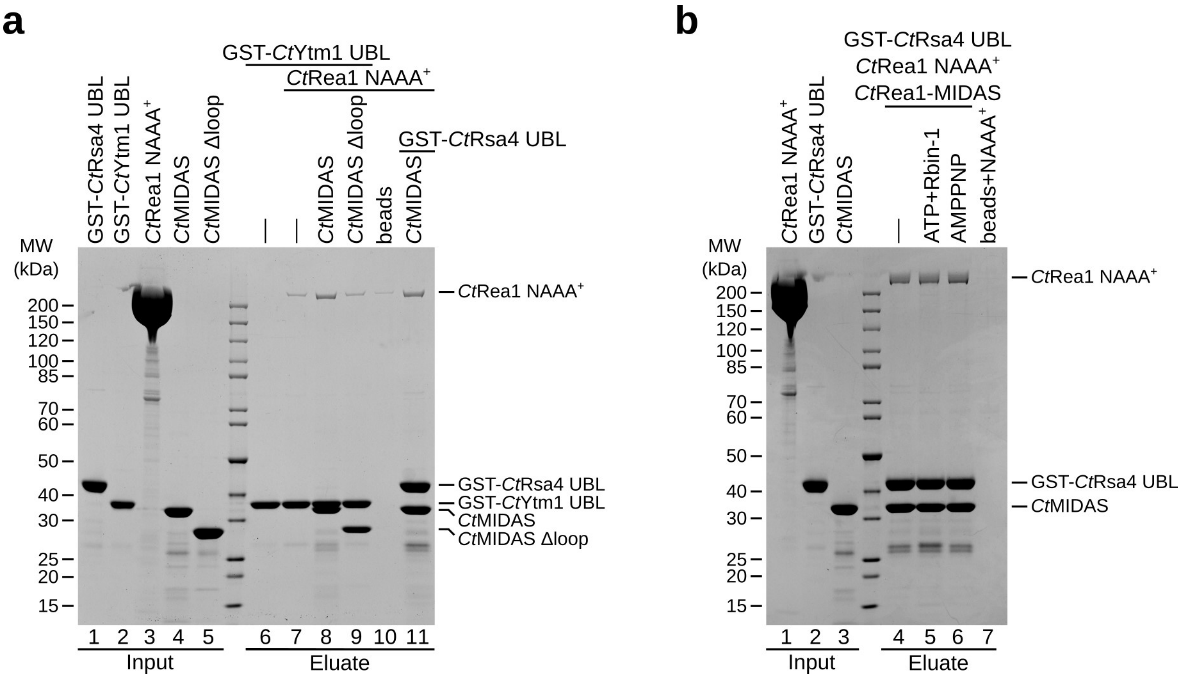

Supplementary Figure 6. The MIDAS element II loop is required for interaction of the Rea1-MIDAS–Ytm1-UBL complex with the Rea1-AAA+ ring. (**a**) GST-tagged *Ct*Ytm1-UBL or *Ct*Rsa4-UBL were incubated with *Ct*Rea1-MIDAS or *Ct*Rea1-MIDASΔloop and the *Ct*Rea1-NAAA+ ring domain. After washing, the GST-tagged *Ct*Ytm1- and *Ct*Rsa4-UBL domains and bound material were eluted with GSH and analyzed with SDS-PAGE and Coomassie staining. Protein inputs are shown in lanes 1–5, final eluates in lanes 6–11. (**b**) Binding assay between GST-tagged *Ct*Rsa4-UBL, *Ct*Rea1-MIDAS and *Ct*-NAAA+ ring domain in the absence of nucleotides, in presence of ATP (2 mM final concentration) and the inhibitor Rbin-1 (0.1 mM final concentration), or in presence of non-hydrolysable AMPPNP (2 mM final concentration). GST-*Ct*Rsa4-UBL and bound material was eluted with GSH and analyzed with SDS-PAGE and Coomassie staining. Protein inputs are shown in lanes 1–3, final eluates in lanes 4–7.

Supplementary Table 1. *E. coli* expression plasmids used in this study

Name	Relevant information	Source
**pET24d-*Ct*MIDAS-(His)$_6$**	Kan^R, T7 promoter, *lac* operator, (aa 4671–4997)	This study
**pETHis-(His)$_6$-*Ct*Ytm1-UBL**	Kan^R, T7 promoter, *lac* operator, (aa 8–98)	This study
**pETHis-(His)$_6$-*Ct*Rsa4-UBL**	Kan^R, T7 promoter, *lac* operator, (aa 31–128)	This study
**pET24d-(His)$_6$-GST-*Ct*Rsa4-UBL**	Kan^R, T7 promoter, *lac* operator, (aa 1–128)	This study
**pET24d-(His)$_6$-GST-*Ct*Ytm1-UBL**	Kan^R, T7 promoter, *lac* operator, (aa 10–98)	This study
**pET-15b-*Ct*MIDAS-(His)$_6$**	Amp^r, T7 promoter, *lac* operator, (aa 4690–4997)	This study
**pET-15b-*Ct*MIDASΔloop-(His)$_6$**	Amp^r, T7 promoter, *lac* operator, (aa 4690–4733-GSG-4774–4997)	This study
**pET24d-GST-*Ct*Kap104**	Kan^R, T7 promoter, *lac* operator	[1]
**pET-15b-*Ct*Syo1-(His)$_6$**	Amp^r, T7 promoter, *lac* operator	[1]
**pET-15b-*Ct*MIDAS PY>A-(His)$_6$**	Amp^r, T7 promoter, *lac* operator, (aa 4690–4997)	This study
**pET-15b-*Ct*MIDAS KK>A-(His)$_6$**	Amp^r, T7 promoter, *lac* operator, (aa 4690–4997)	This study
**pET-15b-*Ct*MIDAS PY/KK>A-(His)$_6$**	Amp^r, T7 promoter, *lac* operator, (aa 4690–4997)	This study

Supplementary Table 2. *S. cerevisiae* plasmids used in this study

Name	Relevant information	Source
**pVA3-1 (p53)**	2µ, *TRP1*, GAL4 BD-murine p53 (aa 72–390), pGBT9	Takara Bio Inc.
**pTD1-1 (SV40)**	2µ, *LEU2*, GAL4 AD-SV40 large T-antigen (aa 84–708), pACT2	Takara Bio Inc.
**YCplac111**	CEN, *LEU2*	[2]
**pGADT7-*Sc*RSA4**	2µ, *LEU2*, P*ADH1*, T*ADH1*, N-terminal GAL4-AD	[3]
**pG4BDC22-Gly-aa4608-*Sc*MIDAS**	CEN, *TRP1*, P*ADH1*, T*ADH1*, C-terminal GAL4-BD	This study
**pG4BDC22-aa4614-*Sc*MIDAS**	CEN, *TRP1*, P*ADH1*, T*ADH1*, C-terminal GAL4-BD	This study
**pG4BDC22-Gly-aa4632-*Sc*MIDAS**	CEN, *TRP1*, P*ADH1*, T*ADH1*, C-terminal GAL4-BD	This study
**pG4BDC22-aa4641-*Sc*MIDAS**	CEN, *TRP1*, P*ADH1*, T*ADH1*, C-terminal GAL4-BD	This study
**pG4BDC22-Gly-aa4655-*Sc*MIDAS**	CEN, *TRP1*, P*ADH1*, T*ADH1*, C-terminal GAL4-BD	This study
**pG4BDC22-aa4683-*Sc*MIDAS**	CEN, *TRP1*, P*ADH1*, T*ADH1*, C-terminal GAL4-BD	This study
**pG4BDC22-aa4696-*Sc*MIDAS**	CEN, *TRP1*, P*ADH1*, T*ADH1*, C-terminal GAL4-BD	This study
**pGADT7-*Ct*RSA4**	2µ, *LEU2*, P*ADH1*, T*ADH1*, N-terminal GAL4-AD	[4]
**pG4BDN22-aa4690-*Ct*MIDAS**	CEN, *TRP1*, P*ADH1*, T*ADH1*, N-terminal GAL4-BD	This study
**pG4BDN22-aa4690-*Ct*MIDAS Δloop**	CEN, *TRP1*, P*ADH1*, T*ADH1*, N-terminal GAL4-BD	This study
**pG4BDN22-aa4690-*Ct*MIDAS DAA**	CEN, *TRP1*, P*ADH1*, T*ADH1*, N-terminal GAL4-BD	This study
**pG4BDN22-aa4690-*Ct*MIDAS Δloop DAA**	CEN, *TRP1*, P*ADH1*, T*ADH1*, N-terminal GAL4-BD	This study
**pG4BDN22-aa4608-*Sc*MIDAS**	CEN, *TRP1*, P*ADH1*, T*ADH1*, N-terminal GAL4-BD	This study
**pG4BDN22-aa4608-*Sc*MIDAS Δloop**	CEN, *TRP1*, P*ADH1*, T*ADH1*, N-terminal GAL4-BD	This study
**pG4BDN22-aa4608-*Sc*MIDAS DAA**	CEN, *TRP1*, P*ADH1*, T*ADH1*, N-terminal GAL4-BD	This study
**pG4BDN22-aa4608-*Sc*MIDAS Δloop DAA**	CEN, *TRP1*, P*ADH1*, T*ADH1*, N-terminal GAL4-BD	This study
**pG4BDN22-aa4690-*Ct*MIDAS F4951R**	CEN, *TRP1*, P*ADH1*, T*ADH1*, N-terminal GAL4-BD	This study
**pG4BDN22-aa4690-*Ct*MIDAS F4951A**	CEN, *TRP1*, P*ADH1*, T*ADH1*, N-terminal GAL4-BD	This study
**pG4BDN22-aa4690-*Ct*MIDAS I4959R**	CEN, *TRP1*, P*ADH1*, T*ADH1*, N-terminal GAL4-BD	This study
**pG4BDN22-aa4690-*Ct*MIDAS I4959A**	CEN, *TRP1*, P*ADH1*, T*ADH1*, N-terminal GAL4-BD	This study
**pG4BDN22-aa4690-*Ct*MIDAS V4956R**	CEN, *TRP1*, P*ADH1*, T*ADH1*, N-terminal GAL4-BD	This study
**pG4BDN22-aa4690-*Ct*MIDAS V4956A**	CEN, *TRP1*, P*ADH1*, T*ADH1*, N-terminal GAL4-BD	This study
**pG4BDN22-aa4690-*Ct*MIDAS V4958R**	CEN, *TRP1*, P*ADH1*, T*ADH1*, N-terminal GAL4-BD	This study
**pG4BDN22-aa4690-*Ct*MIDAS V4958A**	CEN, *TRP1*, P*ADH1*, T*ADH1*, N-terminal GAL4-BD	This study
**pG4BDC22-Gly-aa4608-*Sc*MIDAS Y4859R**	CEN, *TRP1*, P*ADH1*, T*ADH1*, N-terminal GAL4-BD	This study
**pG4BDC22-Gly-aa4608-*Sc*MIDAS Y4859A**	CEN, *TRP1*, P*ADH1*, T*ADH1*, N-terminal GAL4-BD	This study
**pG4BDC22-Gly-aa4608-*Sc*MIDAS I4871R**	CEN, *TRP1*, P*ADH1*, T*ADH1*, N-terminal GAL4-BD	This study
**pG4BDC22-Gly-aa4608-*Sc*MIDAS I4871A**	CEN, *TRP1*, P*ADH1*, T*ADH1*, N-terminal GAL4-BD	This study
**pG4BDC22-Gly-aa4608-*Sc*MIDAS Y4859R I4871R**	CEN, *TRP1*, P*ADH1*, T*ADH1*, N-terminal GAL4-BD	This study
**pG4BDC22-Gly-aa4608-*Sc*MIDAS Y4859A I4871A**	CEN, *TRP1*, P*ADH1*, T*ADH1*, N-terminal GAL4-BD	This study
**YCplac111-P*REA1*-TAP-Flag-*REA1***	CEN, *LEU2*, P*REA1*, T*REA1*, N-terminal TAP-Flag tag	[5]
**YCplac111-P*REA1*-TAP-Flag-*rea1* Y4859A**	CEN, *LEU2*, P*REA1*, T*REA1*, N-terminal TAP-Flag tag	This study
**YCplac111-P*REA1*-TAP-Flag-*rea1* Y4859R**	CEN, *LEU2*, P*REA1*, T*REA1*, N-terminal TAP-Flag tag	This study
**YCplac111-P*REA1*-TAP-Flag-*rea1* I4871A**	CEN, *LEU2*, P*REA1*, T*REA1*, N-terminal TAP-Flag tag	This study
**YCplac111-P*REA1*-TAP-Flag-*rea1* I4871R**	CEN, *LEU2*, P*REA1*, T*REA1*, N-terminal TAP-Flag tag	This study
**YCplac111-P*REA1*-TAP-Flag-*rea1* Y4859A I4871A**	CEN, *LEU2*, P*REA1*, T*REA1*, N-terminal TAP-Flag tag	This study
**YCplac111-P*REA1*-TAP-Flag-*rea1* Y4859R I4871R**	CEN, *LEU2*, P*REA1*, T*REA1*, N-terminal TAP-Flag tag	This study
**pRS314-*RSA4***	CEN, *TRP1*, P*RSA4*, T*RSA4*,	[3]
**pRS314-*rsa4*-1**	CEN, *TRP1*, P*RSA4*, T*RSA4*,	[3]
**pRS314-*YTM1***	CEN, *TRP1*, P*YTM1*, T*YTM1*,	[6]
**pRS314-*ytm1* S78L**	CEN, *TRP1*, P*YTM1*, T*YTM1*,	[6]
**YCplac111-P*REA1*-TAP-Flag-*rea1* ΔMIDAS**	CEN, *LEU2*, P*REA1*, T*REA1*, N-terminal TAP-Flag tag (aa 1–4621)	This study
**YCplac111-P*REA1*-TAP-Flag-*rea1* Δloop**	CEN, *LEU2*, P*REA1*, T*REA1*, N-terminal TAP-Flag tag	This study

Name	Relevant information	Source
**YCplac111-P*REA1*-TAP-Flag-*rea1* DAA**	CEN, *LEU2*, P*REA1*, T*REA1*, N-terminal TAP-Flag tag	[5]
**YCplac111-P*GAL1-10*-TAP-Flag-*REA1***	CEN, *LEU2*, P*REA1*, T*REA1*, N-terminal TAP-Flag tag	[5]
**YCplac111-P*GAL1-10*-TAP-Flag-*rea1* ΔMIDAS**	CEN, *LEU2*, P*GAL1-10*, T*REA1*, N-terminal TAP-Flag tag	This study
**YCplac111-P*GAL1-10*-TAP-Flag-*rea1* Δloop**	CEN, *LEU2*, P*GAL1-10*, T*REA1*, N-terminal TAP-Flag tag	This study
**YCplac111-P*GAL1-10*-TAP-Flag-*rea1* DAA**	CEN, *LEU2*, P*GAL1-10*, T*REA1*, N-terminal TAP-Flag tag	This study
**YCplac111-P*REA1*-GFP-*REA1***	CEN, *LEU2*, P*REA1*, T*REA1*, N-terminal GFP tag	[5]
**YCplac111-P*REA1*-GFP-*rea1* DAA**	CEN, *LEU2*, P*REA1*, T*REA1*, N-terminal GFP tag	[5]
**YCplac111-P*REA1*-GFP-*rea1* ΔMIDAS**	CEN, *LEU2*, P*REA1*, T*REA1*, N-terminal GFP tag	This study
**YCplac111-P*REA1*-GFP-*rea1* Δloop**	CEN, *LEU2*, P*REA1*, T*REA1*, N-terminal GFP tag	This study
**YCplac111-P*ADH1*-*Sc*MIDAS loop-3xGFP**	CEN, *LEU2*, P*ADH1*, T*ADH1*, C-terminal 3xGFP tag	This study
**YCplac111-P*ADH1*-*Ct*MIDAS loop-3xGFP**	CEN, *LEU2*, P*ADH1*, T*ADH1*, C-terminal 3xGFP tag	This study
**YCplac111-P*ADH1*-3xGFP**	CEN, *LEU2*, P*ADH1*, T*ADH1*, C-terminal 3xGFP tag	[1]
**YCplac111-P*ADH1*-*Ct*MIDAS loop PY>A-3xGFP**	CEN, *LEU2*, P*ADH1*, T*ADH1*, C-terminal 3xGFP tag	This study
**YCplac111-P*ADH1*-*Ct*MIDAS loop KK>A-3xGFP**	CEN, *LEU2*, P*ADH1*, T*ADH1*, C-terminal 3xGFP tag	This study
**YCplac111-P*ADH1*-*Ct*MIDAS loop PY/KK>A-3xGFP**	CEN, *LEU2*, P*ADH1*, T*ADH1*, C-terminal 3xGFP tag	This study
**YCplac111-P*ADH1*-*Sc*MIDAS loop PY>A-3xGFP**	CEN, *LEU2*, P*ADH1*, T*ADH1*, C-terminal 3xGFP tag	This study
**YCplac111-P*ADH1*-*Sc*MIDAS loop KR>A-3xGFP**	CEN, *LEU2*, P*ADH1*, T*ADH1*, C-terminal 3xGFP tag	This study
**YCplac111-P*ADH1*-*Sc*MIDAS loop PY/KR>A-3xGFP**	CEN, *LEU2*, P*ADH1*, T*ADH1*, C-terminal 3xGFP tag	This study
**pG4BDN22-aa4608-*Sc*MIDAS loop>L4-PY-NLS**	CEN, *TRP1*, P*ADH1*, T*ADH1*, N-terminal GAL4-BD	This study
**pG4BDN22-aa4608-*Sc*MIDAS loop>Hrp1-PY-NLS**	CEN, *TRP1*, P*ADH1*, T*ADH1*, N-terminal GAL4-BD	This study
**pG4BDN22-aa4608-*Sc*MIDAS loop>Syo1-PY-NLS**	CEN, *TRP1*, P*ADH1*, T*ADH1*, N-terminal GAL4-BD	This study
**YCplac111-P*REA1*-GFP-*rea1* loop>L4-PY-NLS**	CEN, *LEU2*, P*REA1*, T*REA1*, N-terminal GFP tag	This study
**YCplac111-P*REA1*-GFP-*rea1* loop>Syo1-PY-NLS**	CEN, *LEU2*, P*REA1*, T*REA1*, N-terminal GFP tag	This study
**YCplac111-P*REA1*-GFP-*rea1* loop>Hrp1-PY-NLS**	CEN, *LEU2*, P*REA1*, T*REA1*, N-terminal GFP tag	This study
**YCplac111-P*REA1*-TAP-Flag-*rea1* loop>L4-PY-NLS**	CEN, *LEU2*, P*REA1*, T*REA1*, N-terminal TAP-Flag tag	This study
**YCplac111-P*REA1*-TAP-Flag-*rea1* loop>Syo1-PY-NLS**	CEN, *LEU2*, P*REA1*, T*REA1*, N-terminal TAP-Flag tag	This study
**YCplac111-P*REA1*-TAP-Flag-*rea1* loop>Hrp1-PY-NLS**	CEN, *LEU2*, P*REA1*, T*REA1*, N-terminal TAP-Flag tag	This study
**YCplac111-P*GAL1-10*-TAP-Flag-*rea1* loop>L4-PY-NLS**	CEN, *LEU2*, P*GAL1-10*, T*REA1*, N-terminal TAP-Flag tag	This study
**YCplac111-P*GAL1-10*-TAP-Flag-*rea1* loop>Syo1-PY-NLS**	CEN, *LEU2*, P*GAL1-10*, T*REA1*, N-terminal TAP-Flag tag	This study
**YCplac111-P*GAL1-10*-TAP-Flag-*rea1* loop>Hrp1-PY-NLS**	CEN, *LEU2*, P*GAL1-10*, T*REA1*, N-terminal TAP-Flag tag	This study
**YCplac111-P*GAL1-10*-GFP-*REA1***	CEN, *LEU2*, P*GAL1-10*, T*REA1*, N-terminal GFP tag	[5]
**YCplac111-P*GAL1-10*-GFP-*rea1* DAA**	CEN, *LEU2*, P*GAL1-10*, T*REA1*, N-terminal GFP tag	[5]
**YCplac111-*PGAL1-10*-GFP-*rea1* loop>Hrp1-PY-NLS**	CEN, *LEU2*, P*GAL1-10*, T*REA1*, N-terminal GFP tag	This study
**pADH181-ProtA-TEV-NAAA^{+} *Ct*Rea1**	2μ, *LEU2*, P*ADH1*, T*ADH1*, N-terminal ProtA-TEV tag (aa 1–2390)	This study

Supplementary Table 3. *S. cerevisiae* strains used in this study

Name	Relevant genotype	Source
**PJ69-4A**	*trp1-901*, *leu2-3,112*, *ura3-52*, *his3-200*, *gal4Δ*, *gal80Δ*, *LYS2*::*GAL1-HIS3*, *GAL2-ADE2*, *met2*::*GAL7-lacZ*	7
**W303**	Wild type, *MAT*α	8
**DS1-2b ***	Wild type	9
***rea1*Δ shuffle**	*rea1*::kanMX6, pRS416-*REA1*	3
***rea1*Δ *rsa4*Δ shuffle**	*rea1*::kanMX6, *rsa4*::His3MX6, pRS416-*REA1*, pRS316-*RSA4*	3
***rea1*Δ *ytm1*Δ shuffle**	*rea1*::His3MX6, *ytm1*::hphNT1, pRS416-*REA1*, pRS316-*YTM1*	6
**Aid-HA-*REA1***	P_{REA1}-Aid-HA::natNT2, P_{ADH1}-*Os*TIR1-9xmyc::TRP1	This study
***RIX1*-TAP *RPL3*-Flag ***	*RIX1*-TAP::*TRP1*, *RPL3*-Flag::natNT2	5

Supplementary References

1. Kressler, D. *et al.* Synchronizing nuclear import of ribosomal proteins with ribosome assembly. *Science* **338**, 666–671 (2012).
2. Gietz, R. D. & Sugino, A. New yeast-Escherichia coli shuttle vectors constructed with in vitro mutagenized yeast genes lacking six-base pair restriction sites. *Gene* **74**, 527–534 (1988).
3. Ulbrich, C. *et al.* Mechanochemical removal of ribosome biogenesis factors from nascent 60S ribosomal subunits. *Cell* **138**, 911–922 (2009).
4. Baßler, J. *et al.* Interaction network of the ribosome assembly machinery from a eukaryotic thermophile. *Protein Sci. Publ. Protein Soc.* **26**, 327–342 (2017).
5. Matsuo, Y. *et al.* Coupled GTPase and remodelling ATPase activities form a checkpoint for ribosome export. *Nature* **505**, 112–116 (2014).
6. Bassler, J. *et al.* The AAA-ATPase Rea1 drives removal of biogenesis factors during multiple stages of 60S ribosome assembly. *Mol. Cell* **38**, 712–721 (2010).
7. James, P., Halladay, J. & Craig, E. A. Genomic libraries and a host strain designed for highly efficient two-hybrid selection in yeast. *Genetics* **144**, 1425–1436 (1996).
8. Thomas, B. J. & Rothstein, R. Elevated recombination rates in transcriptionally active DNA. *Cell* **56**, 619–630 (1989).
9. Nissan, T. A., Bassler, J., Petfalski, E., Tollervey, D. & Hurt, E. 60S pre-ribosome formation viewed from assembly in the nucleolus until export to the cytoplasm. *EMBO J.* **21**, 5539–5547 (2002).
