# Supplementary figures and images for: Crystal structures of Rea1-MIDAS bound to its ribosome assembly factor ligands resembling integrin–ligand-type complexes
Source: Nat Commun. 2019 Jul 11;10:3050. doi: 10.1038/s41467-019-10922-6 (PMC6624252; doi:10.1038/s41467-019-10922-6)

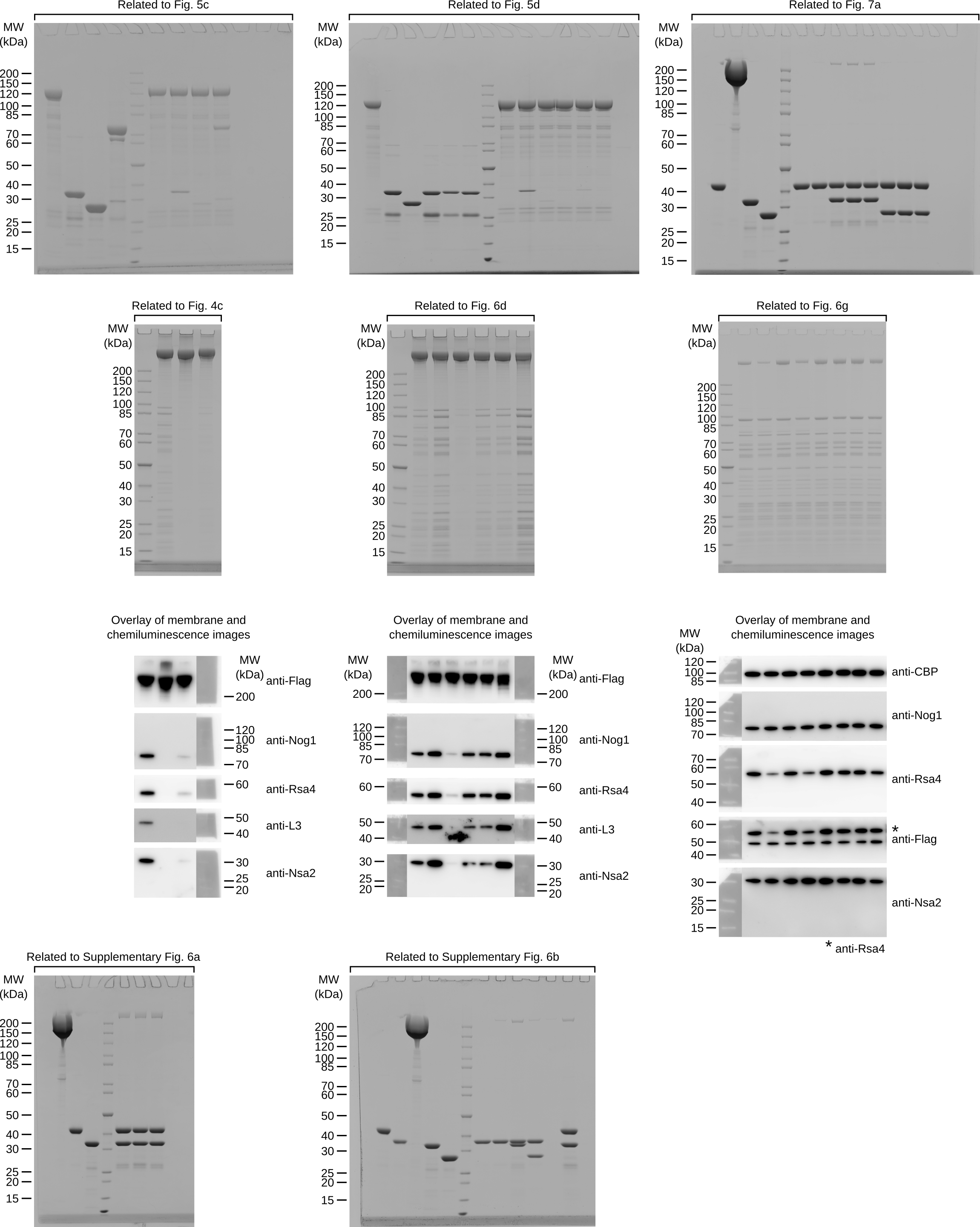

Supplement: Supplementary file 4 — Source Data [file 41467_2019_10922_MOESM4_ESM.png]
